# **Corrigenda: Discovery of the rare genus** Blacometeorus **Tobias, 1976 (Hymenoptera, Braconidae, Blacinae) in the Oriental part of China, with description of a new species**

**DOI:** 10.3897/zookeys.76.915

**Published:** 2011-01-19

**Authors:** Hong-fei Chai, Jun-hua He, Xue-xin Chen

**Affiliations:** State Key Lab of Rice Biology, Institute of Insect Sciences, Zhejiang University, 268 Kaixuan Road, Hangzhou 310029, China

The published figure legend is as follows: (WRONG)

**Figures 1–10.** Blacometeorus sinicus Chai & Chen, sp. n. ♀, holotype. **1** antenna **2** head, frontal view **3** head, dorsal view **4** mesonotum, dorsal aspect **5** propodeum and metasomal tergite I–II, dorsal aspect **6** fore wing **7** hind wing **8** body, lateral view **9** hind leg **10** hind tarsus.

The CORRECT one is as follows:

**Figures 1–10.** Blacometeorus sinicus Chai & Chen, sp. n. ♀, holotype. **1**, head, dorsal view **2** head, frontal view **3** antenna **4** body, lateral view **5** hind leg **6** hind tarsus **7** fore wing **8** hind wing **9** mesonotum, dorsal aspect **10** propodeum and metasomal tergite I–II, dorsal aspect.

